# Changing individual-level risk factors for malaria with declining transmission in southern Zambia: a cross-sectional study

**DOI:** 10.1186/1475-2875-10-324

**Published:** 2011-10-31

**Authors:** Catherine G Sutcliffe, Tamaki Kobayashi, Harry Hamapumbu, Timothy Shields, Aniset Kamanga, Sungano Mharakurwa, Philip E Thuma, Gregory Glass, William J Moss

**Affiliations:** 1 Department of Epidemiology, Bloomberg School of Public Health, Johns Hopkins University, 615 North Wolfe Street, Baltimore, MD, USA; 2 W. Harry Feinstone Department of Molecular Microbiology and Immunology, Bloomberg School of Public Health, Johns Hopkins University, 615 North Wolfe Street, Baltimore, MD, USA; 3 Malaria Research Trust, Choma, Zambia

**Keywords:** malaria, Africa, control strategies, epidemiology

## Abstract

**Background:**

Malaria elimination will require that both symptomatic- and asymptomatic-infected persons be identified and treated. However, well-characterized, individual-level risk factors for malaria may not be valid in regions with declining malaria transmission. Changes in individual-level correlates of malaria infection were evaluated over three years in a region of declining malaria transmission in southern Zambia.

**Methods:**

Malaria surveys were conducted in two study areas within the catchment area of Macha Hospital, Zambia in 2007 and 2008/2009. A random sample of households was identified from a digitized satellite image of the study areas. Cross-sectional surveys were conducted approximately five times throughout the year in each of the two study areas. During study visits, adults and caretakers of children were administered questionnaires and a blood sample was obtained for a rapid diagnostic test (RDT) for malaria.

**Results:**

In the 2007 study area, 330 individuals were surveyed. 40.9% of participants lived in a household with at least one insecticide-treated bed net (ITN); however, only 45.2% reported sleeping under the ITN. 23.9% of participants were RDT positive. Correlates of RDT positivity included younger age, the presence of symptoms, testing during the rainy season, using an open water source, and not sleeping under an ITN. In the 2008 study area, 435 individuals were surveyed. 77.0% of participants lived in a household with at least one ITN; however, only 56.4% reported sleeping under the ITN. 8.1% of participants were RDT positive. RDT positivity was negatively correlated with the presence of symptoms within the last two weeks but positively correlated with documented fever. In 2009, 716 individuals were surveyed in the same area as 2008. 63.7% of participants lived in a household with at least one ITN; however, only 57.7% reported sleeping under the ITN. 1.5% of participants were RDT positive. Only self-reported fever was significantly correlated with RDT positivity.

**Conclusions:**

With declining malaria prevalence, few individual-level characteristics were correlated with RDT positivity. This lack of correlation with individual characteristics hampers identification of individuals infected with malaria. Strategies based on ecological or environmental risk factors may be needed to target control efforts and achieve further reductions and elimination.

## Background

Globally, 225 million cases of malaria and 781, 000 deaths were estimated to occur in 2009, with the majority in sub-Saharan Africa [1]. Recent progress in malaria control resulting from the increased availability and coverage of several interventions, including insecticide-treated bed nets (ITNs), effective artemisinin-based combination therapy, indoor residual spraying of households, and intermittent preventive treatment for pregnant women, is thought to have reduced disease burden. Targets have been set by the United Nations, the World Health Assembly and the Roll Back Malaria Partnership to increase coverage of control measures, reduce the number of malaria cases and deaths by 75% or more by 2015, and eliminate malaria in several countries [1]. With expanded malaria control programmes, several countries in Africa have documented large and sustained decreases in the burden of disease [1].

In regions that have achieved low levels of transmission, further malaria control and elimination will require that interventions are not only incorporated into national control programmes and accepted and used by individuals, but that symptomatic- and asymptomatic-infected persons be identified and treated. Numerous prior studies identified individual-level risk factors for malaria, but many were conducted in areas of high endemicity [2] and among high-risk groups, such as children [3-5] and pregnant women [6,7]. However, in regions with declining malaria transmission as a result of accelerated control efforts, these well-characterized, individual-level risk factors for malaria may not be valid [8].

Zambia is one of eleven countries in sub-Saharan Africa that achieved a greater than 50% reduction in the number of malaria cases between 2000 and 2009 [1]. The prevalence of parasitaemia in children younger than five years of age decreased 53% between the malaria indicator surveys in 2006 and 2008 [9]. Although great spatial heterogeneity remains in the risk of malaria throughout the country, with the highest risk in the northern provinces [10], the Southern Province has witnessed a dramatic decline in the burden of malaria over the past several years [11] and may be a region in which malaria elimination can be achieved [12]. According to a recent Roll Back Malaria Country Report on Zambia, developing an active case-detection system to identify parasites in asymptomatic reservoirs is critical to the development of a malaria elimination programme in Zambia [13]. Such a system requires that communities understand how malaria is transmitted and controlled as well as comply with control programmes, and that high-risk individuals or communities can be identified for targeted interventions. Using a series of cross-sectional studies, the level of general knowledge about malaria, ownership and use of ITNs, and changes in individual-level correlates of malaria parasitaemia were determined over three years in a region of declining malaria transmission in Southern Province, Zambia.

## Methods

### Study site

**Figure 1:**
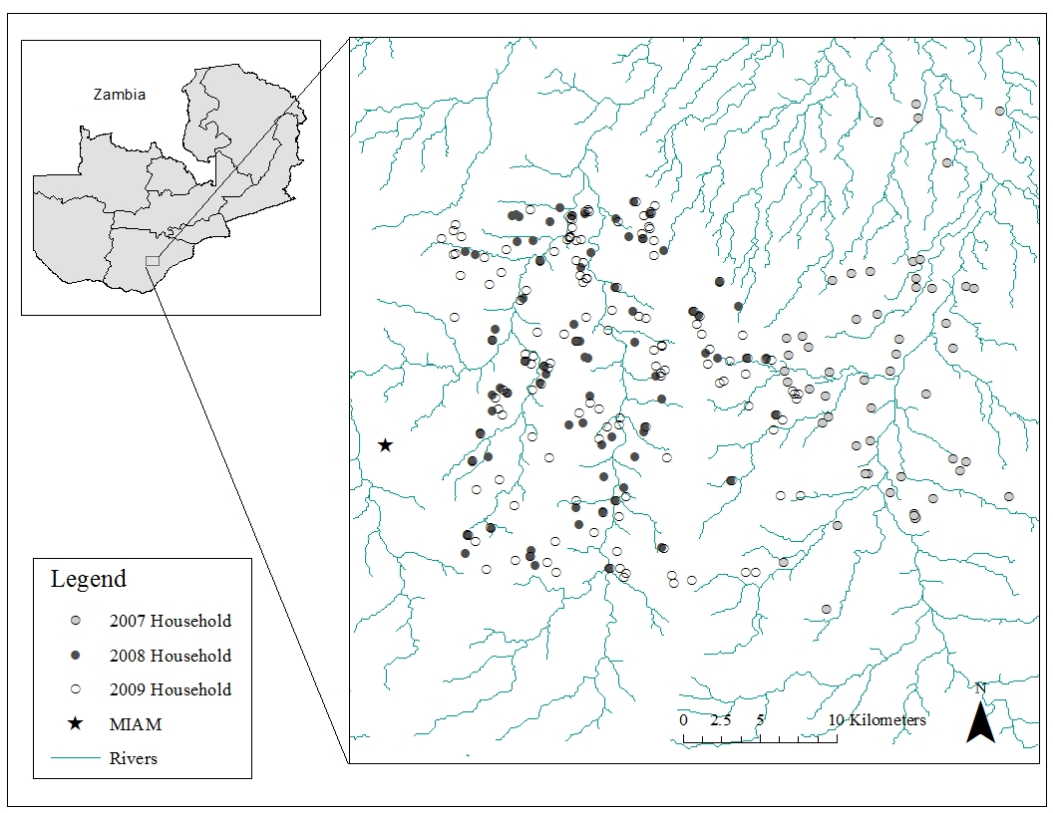
**Map of study sites in Southern Province, Zambia**. Note: The study site in 2007 consisted of a 525 km^2 ^region to the east of Macha Hospital. In 2008 and 2009, the study site was a 575 km^2 ^area west of the 2007 study site and including the hospital and Malaria Institute at Macha.

The study was conducted in the catchment area of Macha Hospital in Choma District, Southern Province, Zambia between April 2007 and December 2009. Macha Hospital is located approximately 70 km from the nearest town of Choma on a plateau at an altitude of approximately 1, 100 m above sea level and in a habitat characterized as Miombo woodland. There is a single rainy season, lasting from approximately November through April, followed by a cool, dry season from April to August, and a hot, dry season from August to November. The catchment area is populated by traditional villagers living in small, scattered homesteads. *Anopheles arabiensis *is the primary vector responsible for malaria transmission [14], which peaks during the rainy season. The study site in 2007 consisted of a 525 km^2 ^region to the east of Macha Hospital and the Malaria Institute at Macha (MIAM; Figure 1). In 2008 and 2009, the study site was shifted for logistical reasons to a 575 km^2 ^area west of the 2007 study site and including the hospital.

The Southern Province of Zambia historically had hyperendemic transmission of *Plasmodium falciparum *[15]. More recently, the entomological inoculation rate (EIR) for *An. arabiensis *was estimated to range from 1.6 to 18.3 infective bites per person per season [14]. Zambia introduced artemether-lumefantrine as anti-malarial therapy in 2002 and ITNs were widely distributed in Southern Province, Zambia in 2007. Widespread indoor residual spraying was not formally conducted in the study area.

### Study methods

Satellite images were used to construct a sampling frame from which households were selected for enrollment into prospective longitudinal and cross-sectional surveys of malaria parasitaemia. A Quickbird™ satellite image was obtained from DigitalGlobe Services, Inc. (Denver, Colorado) and imported into ArcGIS 9.2 (Redlands, CA). Structures of appropriate size and shape were identified as potential residences and locations of households were identified and enumerated manually. Simple random sampling was used to select households for the surveys, which were carried out throughout the year.

Study procedures began with community mobilization activities, including approvals from local chiefs and headmen. A field team was provided with images and coordinates of the randomly selected households. Households consisted of one or more domestic structures where members of a family resided. All individuals within a household were eligible to participate. After obtaining permission from the head of household and individual written informed consent, a questionnaire was administered to each participant older than 18 years of age residing within the household and to parents or guardians of those younger than 18 years of age. Data collected included demographic information, history of recent malaria and anti-malarial treatment, knowledge of malaria transmission and prevention, and the use of ITNs. Each participant's temperature was measured using a Braun Thermoscan^® ^ear thermometer. A blood sample was collected by finger prick for a rapid diagnostic test (RDT) for malaria and haemoglobin determination using a Hemocue^® ^photometer. The RDT (ICT Diagnostics, Cape Town, South Africa) was used to detect *P. falciparum *histidine-rich protein 2. This RDT was shown to detect 82% of test samples with wild-type *P. falciparum *at a concentration of 200 parasites/μL and 98% of test samples with a concentration of 2, 000 parasites/μL, with false positives in 0.6% of negative samples [16]. Individuals who were RDT positive were offered treatment with artemether-lumefantrine (Coartem^®^).

This study was approved by the University of Zambia Research Ethics Committee and by the Institutional Review Board at the Johns Hopkins Bloomberg School of Public Health.

### Statistical methods

All participants in the cross-sectional surveys and the first visit for participants in the longitudinal cohort were included in this analysis. The rainy season was assumed to occur from January 1 to June 30 and November 1 to December 31, and was defined based on rainfall and hospitalization data from Macha Hospital during the study period. Fever was defined as a temperature greater or equal to 38°C. Anaemia was defined by age and sex according to World Health Organization criteria [17]. Individuals were considered anaemic if their haemoglobin was < 11 g/dL and they were 0-4.9 years of age; < 11.5 g/dL and 5-11.9 years of age; < 12 g/dL and 12-14.9 years of age; < 12 g/dL, female and ≥ 15 years of age; < 13 g/dL, male and ≥ 15 years of age.

Characteristics hypothesized to be associated with malaria were compared between individuals who were RDT positive and negative using the chi-square test for binary characteristics and the Wilcoxon ranksum test for continuous characteristics. For years in which multiple correlates of RDT positivity were found, crude and adjusted prevalence ratios were calculated using log-binomial regression with generalized estimating equations to account for clustering by households. Characteristics that were found to be correlated with RDT positivity in the crude analysis (p < 0.10) were included in the adjusted model.

## Results

### Study participants

In 2007, 72 heads of household were approached to participate and 52 (72%) agreed to enroll their households. All of the 330 individuals within the households who were approached agreed to participate. In 2008, 81 heads of household were approached to participate and 76 (94%) agreed to enroll their households. Of the 466 individuals within households who were approached, 435 (93%) agreed to participate. In 2009, 122 heads of household were approached to participate and 119 (98%) agreed to enroll their households. Of the 730 individuals within the households who were approached, 716 (98%) agreed to participate. In all years, the majority of participants were young children and adolescents, and approximately half were male (Table 1). The majority of adults had at least primary school education (Table 1). Households were basic, with the majority using public wells or pumps, pit latrines or no toilet facilities, and lanterns. Most structures were built with earth floors, fired brick or cement walls, and pole and grass roofs (Table 1).

**Table 1:** Characteristics of the study population and households, by study year

	2007	2008	2009
***Participants***	***N = 330***	***N = 435***	***N = 716***

Age: Median (IQR)	12.8 (5.2, 31.8)	14.3 (6.4, 34.3)	13.9 (6.4, 31.5)

0-5	76 (23.0)	84 (19.4)	142 (19.8)

5-10	67 (20.3)	76 (17.6)	124 (17.3)

10-20	63 (19.1)	113 (26.1)	187 (26.1)

20-30	37 (11.2)	36 (8.3)	74 (10.3)

30-40	34 (10.3)	35 (8.1)	63 (8.8)

40-50	23 (7.0)	38 (8.8)	50 (7.0)

50-60	8 (2.4)	25 (5.8)	25 (2.5)

≥ 60	22 (6.7)	26 (6.0)	51 (7.1)

Male: N (%)	153 (46.4)	207 (47.6)	342 (47.8)

Education: N (%)^a^			

< Grade 1	3 (2.3)	7 (3.9)	13 (4.4)

Grade 1-6	29 (22.3)	64 (35.2)	82 (27.6)

Grade 7-12	97 (74.6)	110 (60.4)	197 (66.3)

University/certificate/technical training	1 (0.8)	1 (0.6)	5 (1.7)

***Households ***	***N = 52***	***N = 76***	***N = 119***

Source of water: N (%)			

Private well or pump	5 (9.6)	1 (1.3)	2 (1.7)

Public well or pump	30 (57.7)	39 (51.3)	73 (61.3)

Public stand pipe	0 (0.0)	2 (2.6)	1 (0.8)

Unprotected well	3 (5.8)	12 (15.8)	22 (18.5)

River or stream	14 (26.9)	22 (29.0)	21 (17.7)

Toilet: N (%)			

Pit latrine	24 (46.2)	37 (48.7)	98 (82.4)

No facility/bush/field	27 (51.9)	39 (51.3)	21 (17.6)

Other	1 (1.9)	0 (0.0)	0 (0.0)

Source of light: N (%)			

Candle	4 (7.7)	3 (4.0)	24 (20.2)

Lantern	45 (86.5)	67 (88.2)	77 (64.7)

Other	3 (5.8)	6 (7.9)	18 (15.1)

Material of floor: N (%)			

Cement	18 (34.6)	11 (14.5)	29 (24.4)

Earth	34 (65.4)	65 (85.5)	90 (75.6)

Material of walls: N (%)			

Fired brick/cement	44 (84.6)	69 (90.8)	106 (89.1)

Pole and mud/grass	1 (1.9)	3 (4.0)	9 (7.5)

Unfired bricks	7 (13.5)	4 (5.3)	4 (3.4)

Material of roof: N (%)			

Iron sheets/corrugated tin	12 (23.1)	19 (25.0)	34 (28.6)

Pole and grass	36 (69.2)	56 (73.4)	82 (68.9)

Other	4 (7.7)	1 (1.3)	3 (2.5)

^a ^among participants 18 years of age or older

### General knowledge of malaria

The majority of adults (≥ 18 years) were familiar with the symptoms, causes and prevention of malaria (Table 2). However, a small proportion of participants reported causes of malaria other than mosquito bites related to eating and drinking. In addition, while most participants knew that sleeping under a bed net prevented malaria, few participants were familiar with other personal preventive measures. In the 2007 study area, a quarter of participants reported they did not know the causes of malaria or prevention measures. Fewer reported not knowing causes or prevention measures in the 2008 and 2009 study area.

**Table 2:** General knowledge of malaria symptoms, causes and prevention among participants 18 years or older, by study year

	2007 N = 130	2008 N = 182	2009 N = 297
***Symptoms of malaria***	**N (%)**	**N (%)**	**N (%)**

Fever	71 (54.6)	98 (53.9)	175 (58.9)

Headache	45 (34.6)	101 (55.5)	175 (58.9)

Chills	71 (54.6)	114 (62.6)	197 (66.3)

Vomiting	35 (26.9)	73 (40.1)	111 (37.4)

Diarrhoea	21 (16.2)	40 (22.0)	78 (26.3)

Body ache/pain	14 (10.8)	18 (9.9)	43 (14.5)

Cough	4 (3.1)	27 (14.8)	33 (11.1)

Weakness/fatigue	11 (8.5)	38 (20.9)	46 (15.5)

Flu-like symptoms	1 (0.8)	7 (3.9)	10 (3.4)

Thirsty	4 (3.1)	8 (4.4)	16 (5.4)

Loss of appetite	14 (10.8)	10 (5.5)	25 (8.4)

Yellow eyes/skin	24 (18.5)	19 (10.4)	17 (5.7)

Other	11 (8.5)	13 (7.1)	10 (3.4)

Do not know	14 (10.8)	3 (1.7)	0 (0.0)

***Causes of malaria***			

Mosquito bites	92 (70.8)	152 (83.5)	258 (86.9)

Breathing bad air	0 (0.0)	0 (0.0)	0 (0.0)

Eating bad food	8 (6.2)	8 (4.4)	16 (5.4)

Eating fresh maize	0 (0.0)	6 (3.3)	19 (6.4)

Eating fresh fruits	3 (2.3)	10 (5.5)	16 (5.4)

Eating sugar cane	0 (0.0)	5 (2.8)	14 (4.7)

Drinking bad water	13 (10.0)	18 (9.9)	50 (16.8)

Dirty surroundings	2 (1.5)	12 (6.6)	25 (8.4)

Flies	0 (0.0)	0 (0.0)	2 (0.7)

Other	2 (1.5)	3 (1.7)	7 (2.4)

Do not know	33 (25.4)	24 (13.2)	21 (7.1)

***Preventive measures against malaria***			

Burn a fire in the house	1 (0.8)	0 (0.0)	0 (0.0)

Charms	4 (3.1)	0 (0.0)	0 (0.0)

Do not go outside at certain times of day	0 (0.0)	0 (0.0)	0 (0.0)

Have screens on the windows	0 (0.0)	0 (0.0)	0 (0.0)

Keep skin covered	1 (0.8)	0 (0.0)	1 (0.3)

Sleep under a mosquito net	52 (40.0)	120 (65.9)	227 (76.4)

Spray insecticide inside the house	3 (2.3)	7 (3.9)	5 (1.7)

Take medicine to prevent malaria	8 (6.2)	16 (8.8)	31 (10.4)

Wear insect repellent	0 (0.0)	2 (1.1)	3 (1.0)

Drinking and eating clean water and food	7 (5.4)	12 (6.6)	31 (10.4)

Bury mosquito breeding sites	13 (10.0)	20 (11.0)	20 (6.7)

Keep surroundings clean	11 (8.5)	49 (26.9)	74 (24.9)

Seek early treatment	8 (6.2)	20 (11.0)	39 (13.1)

Other	3 (2.3)	5 (2.8)	11 (3.7)

Do not know	55 (42.3)	30 (16.5)	22 (7.4)

The majority of adults reported learning about malaria from health workers (53.1% in 2007; 53.9% in 2008; 64.0% in 2009). Others learned from posters in health centres (13.1% in 2007; 8.2% in 2008; 2.0% in 2009) and schools (14.6% in 2007; 18.1% in 2008; 13.8% in 2009). Less common sources of information included non-governmental organizations, friends, the radio, and the study team. Some participants reported never having learned about malaria (23.9% in 2007; 11.5% in 2008; 12.5% in 2009). These participants were more likely to be older and to have less than a primary school education.

### ITN use and indoor residual spraying

Less than half of participants in 2007, but the majority of participants in 2008 and 2009, lived in a household with a bed net (Table 3). Most households only had one bed net. The primary reason for not owning a bed net in all three years was cost. Among participants who owned a bed net, only half slept under the bed net. The primary reason for not sleeping under the bed net was that no mosquitoes were around. The proportion of participants sleeping under a bed net was higher among participants surveyed during the rainy season (2007: 68.2%; 2008: 65.6%; 2009: 61.4%). Other common reasons for not sleeping under the bed net included an inability to hang the bed net or sleeping outside, the bed net was old, dirty or needed to be re-treated, and it was too hot under the bed net. Among those sleeping under the bed net, the majority of participants had owned the net for less than two years and few reported ever having treated the bed net. Upon inspection of the households where participants reported sleeping under bed nets, almost all had the bed nets hanging (96.7% in 2007; 100% in 2008; 99.6% in 2009). However, up to half had holes (49.2% in 2007; 11.6% in 2008; 33.1% in 2009). The most common brand of bed net used was long-lasting PermaNet^® ^bed nets (63.9% in 2007; 97.4% in 2008; 99.3% in 2009).

**Table 3:** Use of insecticide treated bed nets, by study years

	2007	2008	2009
***All participants***	***N = 330***	***N = 435***	***N = 716***

Lives in a households with ≥ 1 bed net	135 (40.9)	335 (77.0)	456 (63.7)

1 bed net	92 (27.9)	305 (70.1)	408 (57.0)

2 bed nets	25 (7.6)	25 (5.8)	37 (5.2)

3 bed nets	9 (2.7)	5 (1.2)	8 (1.1)

≥ 4 bed nets	9 (2.7)	0 (0.0)	3 (0.4)

Reasons for not owning a bed net	N = 195	N = 100	N = 260

Cost	135 (69.2)	72 (72.0)	237 (91.2)

Bed nets not available	44 (22.6)	3 (3.0)	9 (3.5)

No knowledge of where to buy one	3 (1.5)	7 (7.0)	3 (1.2)

Not enough for everyone	2 (1.0)	11 (11.0)	0 (0.0)

Not enough space under the bed net	1 (0.5)	0 (0.0)	0 (0.0)

No mosquitoes around	1 (0.5)	0 (0.0)	2 (0.8)

Frequent changes in sleeping places	1 (0.5)	0 (0.0)	4 (1.5)

Too hot under the bed net	0 (0.0)	1 (1.0)	0 (0.0)

Belief that bed nets do not protect against mosquitoes	0 (0.0)	1 (1.0)	2 (0.8)

***Among participants with a bed net***	***N = 135***	***N = 335***	***N = 456***

Sleeps under the bed net	61 (45.2)	189 (56.4)	263 (57.7)

Duration of ownership < 2 years	25 (41.0)	175 (92.6)	207 (78.7)

History of treating bed net	13 (21.3)	49 (25.9)	48 (18.3)

Reasons for not sleeping under a bed net	N = 74	N = 146	N = 193

No mosquitoes around	51 (68.9)	55 (37.7)	89 (46.1)

Inability to hang the bed net/sleeping outside	3 (4.1)	111 (76.0)	9 (4.7)

Bed net is old/dirty/needs to be re-treated	4 (5.4)	21 (14.4)	25 (13.0)

Frequent changes in sleeping places	8 (10.8)	17 (11.6)	18 (9.3)

Too hot under the bed net	4 (5.4)	37 (25.3)	29 (15.0)

Not enough space under the bed net	5 (6.8)	0 (0.0)	8 (4.2)

Not the rainy season	1 (1.4)	7 (4.8)	0 (0.0)

Bed net is itchy	0 (0.0)	5 (3.4)	0 (0.0)

Belief that bed nets do not protect against mosquitoes	0 (0.0)	7 (4.8)	11 (5.7)

Only 1.8% of participants in 2007, 1.4% in 2008 and 2.0% 2009 reported that their house had ever been sprayed with insecticide to control mosquitoes.

### Prevalence of malaria, reported symptoms, and correlates of RDT positivity

**Figure 2:**
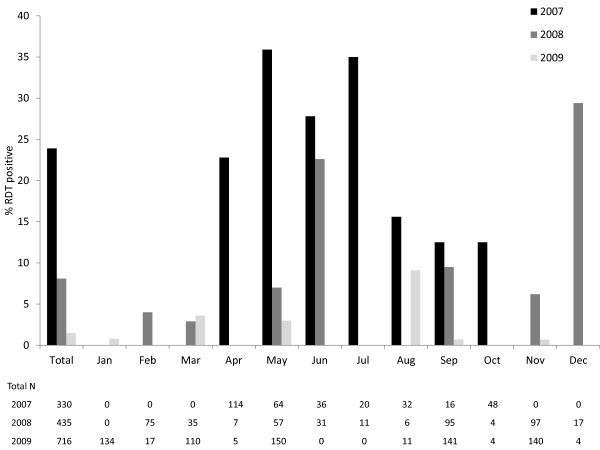
**Prevalence of malaria by month and year in Southern Province, Zambia**.

The prevalence and correlates of RDT positivity differed by study location and year. In the 2007 study area, 79 (23.9%) participants were positive for malaria by RDT (Figure 2). RDT positive individuals were significantly more likely to report any symptoms of malaria in the prior 48 hours, including fever, headache, chills, diarrhoea, nausea/vomiting, and cough, and to report multiple symptoms (see Additional file 1). 51.9% of RDT positive individuals reported a fever in the prior 48 hours, although only 10.1% had a fever as measured on the day of the study visit. The majority of fevers were documented among infected individuals 5-10 years of age. RDT positivity was also significantly correlated with younger age, not sleeping under a bed net, having a study visit in the rainy season, and using an open water source (see Additional file 1). RDT positivity was marginally positively correlated with the presence of anaemia. Symptoms of malaria, younger age, and using an open water source remained significantly independently correlated with RDT positivity after adjustment for other factors (Table 4). Not sleeping under a bed net and having a study visit in the rainy season were marginally independently correlated with RDT positivity.

**Table 4:** Prevalence ratios for correlates of RDT positivity among participants in 2007

Characteristic	Crude prevalence ratio (95% CI)	Adjusted prevalence ratio (95% CI)
Age in years	0.98 (0.97, 0.99)	0.98 (0.97, 0.99)

Any symptoms in prior 48 hours	1.94 (1.29, 2.93)	2.06 (1.33, 3.17)

Sleeps under a bed net	0.50 (0.24, 1.03)	0.50 (0.25, 1.02)

Rainy season	1.60 (0.85, 2.99)	1.67 (1.00, 2.80)

Presence of anaemia	1.45 (1.02, 2.06)	1.27 (0.92, 1.75)

Open water source	1.72 (1.04, 2.85)	1.74 (1.14, 2.64)

In the 2008 study area, 35 (8.1%) participants were positive for malaria by RDT (Figure 2). Unexpectedly, RDT positive individuals were less likely to report symptoms of malaria, particularly in the prior two weeks (see Additional file 1). However, RDT positive individuals were significantly more likely to have a fever as measured on the day of the study visit. The majority of fevers were documented among infected individuals 10-15 years of age. No other characteristics correlated with RDT positivity.

In 2009, 11 (1.5%) participants were positive for malaria by RDT (Figure 2). RDT-positive individuals were marginally more likely to report any symptoms of malaria in the prior 48 hours and to report multiple symptoms (see Additional file 1). 54.6% of RDT positive individuals reported a fever in the prior 48 hours but none had a fever on the day of the study visit. RDT positivity was also marginally positively correlated with the presence of anemia.

## Discussion

In this series of cross-sectional studies in southern Zambia, knowledge of the symptoms, causes and prevention of malaria was high in the two study areas over three calendar years. The prevalence of malaria varied over the study period, from 23.9% in the 2007 study area to 8.1% and 1.5% in the 2008 and 2009 study area. Most infected individuals reported symptoms of malaria, although few had documented fever at the time of the study visit. In the first study area, when the prevalence of malaria was highest, several established characteristics were correlated with RDT positivity, including young age, reported symptoms, documented fever, failure to sleep under a bed net, rainy season and use of open water sources. In the second and third study years, with markedly lower malaria prevalence, few individual-level characteristics were correlated with RDT positivity.

Ascertaining and improving general knowledge of malaria is crucial to the acceptance and uptake of prevention practices in a community. The majority of participants in all years correctly identified the symptoms of malaria and knew that malaria was transmitted by mosquitoes. Similar to other regions [18], most participants acquired knowledge of malaria from health workers or at school. However, a considerable proportion of participants either did not report the correct cause of malaria or, as in other studies in the region [19-21], identified other causes primarily related to drinking and eating food. This lack of accurate knowledge regarding causes of malaria was reflected in the level of knowledge regarding preventive measures, particularly in 2007 when ITN distribution began in the area. As few as 40% of participants reported bed nets as a prevention measure, few other measures were identified, and many participants reported they did not know how to prevent malaria. The survey instrument was based on an allopathic concept of malaria and further ethnographic research is needed to explore the local taxonomy of illness and explanatory models prevalent in the community.

Bed net ownership varied from 40.9% to 77.0%; however, in all study years, approximately half of participants who owned an ITN did not sleep under it. The primary reason provided for not sleeping under a bed net was the absence of mosquitoes, suggesting seasonal use of ITN. Indeed, participants were more likely to report sleeping under an ITN during the rainy season, as reported in other studies [21]. Other reported reasons for not sleeping under the bed nets were primarily logistical [19,22]. These included an inability to hang the bed net or sleeping outside, which was particularly problematic for participants in 2008, changing sleeping places, difficulties sleeping under a bed net due to heat, crowding, or discomfort, and the bed nets being old or dirty. These factors and their impact on ITN use and subsequent protection from malaria infection need to be considered in control programmes to maximize the benefits of ITN.

The prevalence of malaria ranged from 23.9% in 2007 to 1.5% in 2009 in this study conducted over three calendar years and two geographical areas. In 2007, when transmission was highest, several well established risk factors correlated with infection, including younger age [23], presence of anaemia [24], using open water sources [4], sleeping without a bed net [3-5,8], rainy season [5,7], and self-reported or documented symptoms [2]. However, in 2008 and 2009, when the prevalence of malaria was substantially lower, only self-reported or documented fever correlated with infection. With declining prevalence, malaria was no longer likely to be diagnosed in the rainy season or among individuals sleeping without a bed net. The median age of infected individuals increased from 9.0 years with high parasite prevalence to 12.5 and 13.2 years with low prevalence, and was no longer significantly different from the age of the general study population. This shift to higher age groups with declining transmission has been observed in other studies and settings [8,23,25]. Although the power to detect differences decreased with declining malaria prevalence, many characteristics correlated with RDT positivity in 2007 were not associated with RDT positivity in 2008 and 2009.

Only symptoms, particularly fever, correlated with RDT positivity in the time periods with low levels of parasite prevalence. When all symptoms of malaria were considered together, infected individuals were more likely to report symptoms and in greater numbers in 2007 and again in 2009. However, in all years, symptoms of malaria were highly prevalent in RDT-negative individuals, with up to 63% of RDT-negative individuals reporting symptoms within the 48 hours prior to the study visit, thus decreasing the specificity of symptoms in identifying infected persons. RDT-positive individuals were more likely to have documented fever in 2007 and 2008 but only 10-20% of positive individuals had fever. Most RDT-positive individuals were without fever at the time of testing. Other studies in various transmission settings found a large proportion (up to 96%) of individuals infected with malaria to be asymptomatic [26-28]. This finding poses a significant challenge to malaria elimination as many control strategies rely on the identification and treatment of symptomatic individuals seeking care at health centres [29].

This study was subject to several limitations. First, the study was conducted over two geographic areas. Participants surveyed in 2008 and 2009 were from the same geographic area, while those surveyed in 2007 were from an area further east. Consequently, it is possible that the different risk factors found between the two areas, was not due to the decline in malaria transmission but to differences in the ecology or characteristics and behaviours of the participants surveyed. When participants and households were compared, however, there did not appear to be many differences based on measured characteristics. Second, we did not collect information on behaviors, occupation, assets, migration, and travel that might be associated with malaria. Finally, as previously discussed, the power to detect associations was low in 2008 and 2009 with declining malaria transmission.

## Conclusions

In an area with high malaria prevalence, several established, individual-level characteristics were correlated with RDT positivity. With lower malaria prevalence, however, few individual-level characteristics were correlated with RDT positivity and the majority of infected persons were asymptomatic. This lack of correlation with previously established individual-level characteristics hampers identification of asymptomatic-infected individuals critical to the development of a malaria elimination programme in Zambia [13]. Strategies based on ecological or environmental risk factors [30] may be needed to target control efforts and achieve further reductions and elimination in southern Zambia.

## List of abbreviations

EIR: entomological inoculation rate; IQR: inter-quartile range; ITN: insecticide-treated net; RDT: rapid diagnostic test.

## Competing interests

The authors declare that they have no competing interests.

## Authors' contributions

CGS performed the data analysis and drafted the manuscript. TK participated in the coordination of the study and reviewed the manuscript. HH participated in the design and coordination of field aspects of the study and reviewed the manuscript. TS participated in the design and coordination of the study and reviewed the manuscript. AK participated in the design and coordination of field aspects of the study and reviewed the manuscript. SM participated in the design and coordination of the study and reviewed the manuscript. PET participated in the design and coordination of the study and reviewed the manuscript. GG participated in the design and coordination of the study and reviewed the manuscript. WJM conceived of the study, participated in its design and coordination, and participated in the preparation of the manuscript. All authors read and approved the final manuscript.

## Supplementary Material

Additional file 1 **Correlates of RDT positivity, by study year**. ^a ^among participants 18 years of age or older ^b ^self-reported symptoms include fever, headache, chills, nausea/vomiting, cough, diarrhea ^c ^anemia defined by age and sex: < 11 g/dL for children 0-4.9 yrs; < 11.5 g/dL for children 5-11.9 yrs; < 12 g/dL for children 12-14.9 yrs; < 12 g/dL for women ≥ 15 yrs; < 13 g/dL for men ≥ 15 yrs ^d ^open water source: unprotected well or river/stream. Click here for file
